# Workshop on Carbohydrate Moieties as Vaccine Candidates

**DOI:** 10.3201/eid1104.041142

**Published:** 2005-04

**Authors:** Christopher E. Taylor

**Affiliations:** * National Institutes Health, Bethesda, Maryland, USA

**Keywords:** Polysaccharide vaccine, immune response, carbohydrate microarrays

**Workshop on Carbohydrate Moieties as Vaccine Candidates**

Bethesda, MD, USA

October 6-7, 2004

Whole microbes, microbial subunits and extracts, and peptide and protein antigens have been the focus of much vaccine research and development. While studies of peptide and protein antigens have been facilitated by the rapid advances in genomics and proteomics, studies of sugar chains, which are abundantly expressed on the outer surfaces of viral, bacterial, protozoan, and fungal pathogens and on the membranes of mammalian cells, have not kept pace with technologic advances. Polysaccharide-based vaccines have demonstrated efficacy in disease-prevention strategies, e.g., *Haemophilus influenzae* b and pneumococcal conjugate vaccines. However, our understanding of several aspects of polysaccharide vaccines is limited, and more knowledge is needed to allow greater development and deployment. The goals of this workshop were to examine the mechanisms involved in generating an appropriate immune response to selected carbohydrate antigens, highlight recent and novel advances, and discuss how this information could be used in the development of effective vaccines. The workshop participants included national and international research scientists and clinicians from the National Institutes of Health, the Food and Drug Administration, academia, and industry.

The meeting was organized into 7 sessions on such topics as genetic and cellular mechanisms of carbohydrate immunity, carbohydrate antigens for vaccines, and new tools for studying carbohydrates. Understanding the mechanistic aspects of the genetic control and the cellular pathways of the immune response to bacterial carbohydrate antigens should provide insights into ways to enhance the immune response and thus facilitate vaccine development. Studies were also presented on novel molecules involved in the recognition of carbohydrate antigens such as specific intercellular adhesion molecule (ICAM)–grabbing nonintegrins, which are C-type lectins that show substantial expression in many tissues, and toll-like receptors, which function as pattern recognition receptors for conserved pathogen structures and serve as key links between innate and adaptive immunity. Investigations are ongoing to determine how these molecules function in bacterial clearance and in signaling innate and adaptive responses. A number of presentations were focused on the role of CD1 proteins, which present lipid antigens (e.g., from mycobacteria or *Francisella tularensis*, a potential weapon of bioterrorism) to T cells. The evidence that CD1-restricted T cells contribute to immunity against microbial infection includes the observation that CD1 is expressed at higher levels in lesions of tuberculoid leprosy in comparison to lepromatous leprosy. The design of optimal vaccines against such pathogens should include lipid and peptide antigens.

Presentations from several invited experts emphasized the current challenges facing the development of vaccines for meningococcal meningitis. Given that group B meningococcal capsular polysaccharide is similar to host molecules, studies are ongoing to identify vaccine candidates that elicit protective antibody without eliciting autoantibodies. A licensed outer membrane vesicle vaccine was recently introduced for widespread use in New Zealand to control an epidemic. Regarding groups A and C polysaccharides, differences exist in age-related immune responses; for example, for unknown reasons group A polysaccharide is uniquely immunogenic in infants as young as 6 months of age and repeated doses elicit booster antibody responses, whereas group C is poorly immunogenic and repeated doses do not induce adequate responses.

A continuing problem in vaccine research is devising methods to enhance the immune response and then ensuring that it is protective against disease; for instance, the use of CD40 agonist antibody plus antigen showed remarkably rapid immune response and protection in a murine model of anthrax. Equally challenging are efforts to develop combined and multivalent vaccines; several new approaches were discussed. For instance, a diphtheria, tetanus, pertussis, hepatitis B, *H. influenzae* b, meningococcal A and C combination vaccine has been tested in an open, randomized, controlled study. It induces groups A and C bactericidal antibody responses in infants and has reactogenicity profiles similar to meningococcal A and C conjugates given separately. Recent efforts in HIV vaccine research indicate that producing a multivalent envelope glycan conjugate vaccine to induce production of broad neutralizing antibodies is possible.

New technologies such as carbohydrate microarrays, automated syntheses of oligosaccharides, and biophysical and computational methods for studying antigen-antibody interactions are now available for providing insight into the structure of and immune response to carbohydrate antigens. Additionally, peptide mimotopes may be used in carbohydrate technologies designed for proteins. An oligosaccharide synthesizer is now being used in the development of a number of vaccines, including those for malaria, leishmaniasis, HIV, tuberculosis, and leprosy. A Consortium for Functional Genomics (Web site available from http://www.functionalglycomics.org) has been established at the Scripps Research Institute and has developed a novel glycan array format that uses covalent coupling of glycans to glass slides.

At the conclusion of the workshop, participants were asked to identify gaps in knowledge and resource needs. The gaps include 1) elucidating the mechanisms of immunity to and regulation of carbohydrate antigens in adults, children, and neonates and using opportunities (e.g., computer capacity) for modeling carbohydrate antigen-antibody interactions; 2) defining the molecular basis of enhanced immunogenicity with glycoconjugate vaccines and investigating the role of adjuvants; 3) examining the role of CD1-reactive T cells in the immune response to capsular polysaccharides; and 4) developing surrogates for in vivo immunity for the use of glycolipids as CD1-based vaccines in humans. Resource needs include the following: 1) enhanced availability of carbohydrate microarrays; 2) development of appropriate animal models and the availability of a transgenic mouse platform that allows generation of human antibodies; 3) development of tetramers and analytical chemistry to help in the identification of antigens, e.g., for the use of glycolipids as CD-1-based vaccines; and 4) enhanced good laboratory practice (GLP) resources to produce synthetic carbohydrate vaccines and GLP testing for vaccine candidate safety in animals and for production of good manufacturing practice vaccine candidates. As a result of the workshop, an open LISTSERV (GLYCOIMMUNOLOGY) has been established, and the Journal of Clinical Infectious Diseases will publish a review article. For additional information, contact ctaylor@niaid.nih.gov

The workshop was sponsored by funds from the National Vaccine Program Office and the National Institute of Allergy and Infectious Diseases.

